# Human Babesiosis, Bolivia, 2013

**DOI:** 10.3201/eid2208.150195

**Published:** 2016-08

**Authors:** Simona Gabrielli, Valentina Totino, Fabio Macchioni, Freddy Zuñiga, Patricia Rojas, Yuni Lara, Mimmo Roselli, Alessandro Bartoloni, Gabriella Cancrini

**Affiliations:** Università “Sapienza” di Roma, Rome, Italy (S. Gabrielli, V. Totino, G. Cancrini);; Università degli Studi di Pisa, Pisa, Italy (F. Macchioni);; Distrito de Salud Cordillera, Santa Cruz, Bolivia (F. Zuñiga, P. Rojas);; Hospital S. Antonio de Los Sauces, Monteagudo, Bolivia (Y. Lara);; Università di Firenze, Florence, Italy (M. Roselli, A. Bartoloni)

**Keywords:** Babesia microti, humans, Bolivia, ticks, vector-borne infections, parasites

## Abstract

To investigate human babesiosis in the Bolivian Chaco, in 2013 we tested blood samples from 271 healthy persons living in 2 rural communities in this region. Microscopy and PCR indicated that 3.3% of persons were positive for *Babesia microti* parasites (US lineage); seroprevalence was 45.7%. Appropriate screening should mitigate the risk for transfusion-associated babesiosis.

Babesiosis is an emerging tickborne disease worldwide resulting from ≈100 *Babesia* parasite species that can infect a broad range of hosts in which it induces malaria-like disorders. Like trypanosomes and *Plasmodium* spp., *Babesia* spp. can be transmitted through vertical routes and blood transfusions (*1*). Babesiosis has the greatest effect on the cattle industry and on companion animals; however, occasional human babesiosis has attracted increased attention. In immunocompetent persons, the infection is rarely detected because it can be asymptomatic or cause mild, self-resolving symptoms. However, babesiosis can be life-threatening in certain populations, such as neonates/infants, elderly persons, asplenic patients, and otherwise immunocompromised persons (*2*).

The predominant species that causes of human babesiosis in the United States and is a rare cause of disease in Europe and Asia is *Babesia microti* (*3*), a complex that includes at least 4 named types and an unknown number of other strains (*4*). The second-most important zoonotic species, *B. divergens*, causes several clinical cases in Europe (*5*). Other species, such as *B. duncani*, *B. venatorum*, and some *B. divergens*–like parasites, can cause further human infections worldwide (*6*). Many ixodid tick species can transmit *Babesia* parasites to their natural hosts; *Ixodes scapularis* and *I. ricinus* are the most important human-biting vector ticks in the United States and Europe, respectively (*7*).

Three cases of uncharacterized babesiosis have been reported from South America, 2 from Brazil and 1 from Colombia (*8*,*9*). In Bolivia, only cattle have been investigated, highlighting the prevalence of the species *B. bovis* and *B. bigemina*, unstable and endemic in the east of the country (*10*); no data are available on *B. microti*. Our objective was to investigate human babesiosis in the Bolivian Chaco, a rural region in southeastern Bolivia.

## The Study

In 2013, a total of 271 healthy volunteers, residents of 2 rural communities in southeastern Bolivia, Bartolo (Hernando Siles Province, Department of Chuquisaca) and Ivamirapinta (Cordillera Province, Department of Santa Cruz), completed a questionnaire interview asking for anamnestic data (sex, age, fever attacks, history of tick bite or transfusion) and provided blood samples for testing. The participants represented ≈50% and ≈25% of the population of the 2 communities, respectively. The Bolivian Ministry of Health and the Regional Health Departments approved the study design, including its ethical aspects; the Guaraní political organization (Asamblea del Pueblo Guaraní) supported the field work and conducted the interviews.

Blood drawn was immediately used to prepare thick and thin smears and to impregnate filter papers (100 μL); serum was obtained from each remaining sample. Smears were Giemsa stained and examined by microscopy at 400× and 1,000×. DNA was extracted from all the dried blood spots by using Dried Blood Spot DNA Isolation Kit (Norgen Biotek Corp., Thorold, ON, Canada) and amplified by PCR with generic apicomplexan 18S rRNA-specific primers (*11*). Amplicons (≈1,700 bp) were purified (Sure Clean kit; Bioline, Rome, Italy) and then sequenced. Sequences were aligned and compared with those available in GenBank. To investigate the *B. microti* strain, we further examined all positive samples by lineage-specific PCR based on the subunit 7 (η) of the chaperonin-containing t-complex polypeptide 1 (CCTη) gene, following the published protocol (*4*). PCR-positive samples and further randomly chosen serum samples (n = 47 for each community) were checked by an indirect fluorescent antibody test (IFAT) (IgG IFA kit; Fuller Laboratories, Fullerton, CA, USA) for reactivity to *B. microti*, following the manufacturer’s instructions and fixing the cutoff value at dilution 1:64. Positive and negative control sera supplied by the kit were included on each IFAT slide. IFAT sensitivity and specificity in detecting *B. microti* antibodies, reported by the kit data sheet, are 88%–96% and 90%–100%, respectively (*12*). To ensure the specificity of the results, we further tested all reactive serum samples and 10 negative serum samples with the Falciparum-Spot IF kit (bioMérieux, Marcy l’Etoile, France) to detect plasmodial antibodies.

We conducted statistical analyses using the χ^2^ test. We considered p<0.05 as significant.

Of the 271 serum samples, 9 (3.3%; 95% CI 0.97%–5.03%) thin and thick smears, from 5 (4.1%) and 4 (2.7%) participants living in Bartolo and Ivamirapinta, respectively, were positive for *B. microti* (Table; Figure). Infection rates did not differ significantly by community (p = 0.55), sex, or age, despite early infection in Bartolo.

**Table:** *Babesia microti* results from microscopy, PCR, and serology in persons living in 2 rural communities, southeastern Bolivia, 2013

Age group, y	No. positive/no. examined (%)				
	Bartolo			Ivamirapinta	
	Microscopy and PCR	IFAT		Microscopy and PCR	IFAT
1–10	2/29 (6.9)	3/9 (33.3)		1/21(4.7)	5/6 (83.3)
11–20	1/16 (6.2)	1/3 (33.3)		0/42 (0)	3/10 (30)
21–30	1/23 (4.3)	7/7 (100)		1/15 (6.6)	1/3 (33.3)
31–40	0/19 (0)	3/4 (75)		1/21 (4.7)	2/3 (66.6)
41–50	0/11 (0)	0/2 (0)		0/13 (0)	3/4 (75)
51–60	1/11 (9.0)	2/8 (25)		0/13 (0)	2/6 (33)
61–70	0/10 (0)	5/10 (50.0)		1/15 (6.6)	4/11 (36.4)
>70	0/4 (0)	1/4 (25)		0/8 (0)	1/4 (25)
Total	5/123 (4.1)	22/47 (46.8)		4/148 (2.7)	21/47 (44.7)

*IFAT, indirect fluorescent antibody test.

**Figure:**
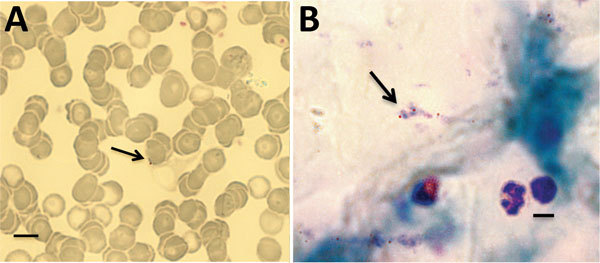
*Babesia microti* parasites (arrows) detected in Giemsa-stained thin (A) and thick (B) blood smears from persons living in 2 rural communities, southeastern Bolivia, 2013. Scale bars indicate 10μm.

Testing of blood from the 9 positive participants by molecular amplification and sequencing confirmed morphologic diagnosis (99% nt identity with the *B. microti* 18S rRNA gene; GenBank accession no. AY693840.1); moreover, results classified all the remaining blood spots as negative. PCR on the CCTη gene and sequencing showed that all positive samples belonged to the US lineage Gray strain (100% nt identity with GenBank accession no. AB362586.1). Sequences obtained were deposited in GenBank under accession nos. KT318131, KT318132, and KT844553–KT844568.

IFAT showed reactivity to *B. microti* antigens in all positive survey participants and in 34 of 85 additional persons, providing an overall seroprevalence of 45.7% (95% CI 35.7%–55.7%). We observed no differences between the 2 communities (p = 0.836) by age group or between early (0–30-year-olds) or late (>30-year-olds) developed seroreactivity (Bartolo: p = 0.209; Ivamirapinta: p = 0.760). We found no cross-reactivity to plasmodial antigens.

## Conclusions

Although our study has some limitations, including the cross-sectional design, the limited number of human samples, and the nonrandom sampling, we detected *B. microti* antigens in ≈3% of persons living in the rural communities of the Bolivian Chaco. Moreover, we detected an overall seroprevalence rate of ≈45%, higher than that reported in Colombia (30.6%) (*13*), and with exposure starting from an early age. None of the positive study participants had signs or symptoms of babesiosis at the time of sample collection. Considering that many intra-erythrocytic cycles are needed before the immune system responds to the parasite and starts antibody production (*14*), the contemporary detection in some cases of blood parasite and serum-specific antibodies suggests a late infection stage.

The presence of *B. microti* antigens in persons without a history of tick infestation or transfusions indicates that contact between ticks and humans is not rare (mainly in young persons), as confirmed by a serosurvey that evidenced human exposure to other tickborne pathogens, such as *Borrelia* spp. (*15*). Furthermore, this finding suggests that, even though the primary reservoir, the white-footed mouse (*Peromyscus leucopus*), has been reported only in North and Central America, natural animal hosts of this protozoon are widespread in the studied area. Inhabitants of both communities live in close contact with domestic animals, such as dogs, chickens, and pigs, and with deer and other wild animals, which might contribute to the maintenance and spread of the ticks. Because this zoonotic babesiosis is due to the Gray strain, previously documented in humans in the United States, Germany, Russia, China, South Korea, and Japan, where it is harbored by various small mammals, further studies are needed to explore its vectors and reservoirs in the rural areas here investigated.

Human babesiosis is probably an underestimated health problem in the Bolivian Chaco. Residents should therefore be alerted to the threat posed by ticks, and physicians should be aware of infection with *B. microti* parasites as a potential life-threatening disease. The presence of *B. microti* antigens in the blood of asymptomatic persons is of concern in terms of the possible risk for transfusion-associated babesiosis and should prompt the need to evaluate implementation of appropriate screening measures.
